# Noncommunicable diseases, access to essential medicines and universal health coverage

**DOI:** 10.1080/16549716.2019.1670014

**Published:** 2019-10-01

**Authors:** David Beran, Hanne Bak Pedersen, Jane Robertson

**Affiliations:** a Division of Tropical and Humanitarian Medicine, University of Geneva and Geneva University Hospitals, Geneva, Switzerland; b Health Technologies and Pharmaceuticals Programme, WHO Regional Office for Europe, Copenhagen, Denmark; c Consultant to World Health Organization Regional Ofﬁce for Europe, Copenhagen, Denmark, Conjoint Senior Lecturer, Clinical Pharmacology, The University of Newcastle, Callaghan, Australia

**Keywords:** Universal health coverage, medicines, access, health systems

## Abstract

Universal Health Coverage is key to reach the overall health-related Sustainable Development Goal, and within this, access to safe, effective, quality, and affordable essential medicines is critical. Currently, medicines for noncommunicable diseases in many countries are not available when needed and if they are present, are unaffordable. Countries face the challenges of rising prevalence of noncommunicable diseases due to increasing risk factors and ageing populations, along with under-diagnosis and under-treatment. Providing noncommunicable disease medicines is only one piece of a complex picture of providing care within Universal Health Coverage that requires strengthening health-care systems, as well as financial resources, priority setting, and monitoring and evaluation systems. Financing for Universal Health Coverage needs to enable adequate resources to be allocated for medicines with a focus on equity as well as priority setting for noncommunicable diseases medicines for reimbursement in benefits packages, efficient procurement and distribution of these medicines, supported by price regulation. These processes need to be evidence-based, transparent and grounded on national values and priorities. Monitoring and evaluation of availability and affordability are key components of sustainable reimbursement systems. With the current Universal Health Coverage agenda, the World Health Organization and countries can no longer ignore the issue of access to medicines for noncommunicable disease and need to develop the appropriate responses in order to guarantee equitable access.

## Background

Noncommunicable diseases (NCD) including diabetes, cardiovascular diseases (CVD), chronic respiratory diseases (CRD) and cancer are the leading causes of mortality worldwide [1]. Deaths due to NCDs represented 72.3% of total deaths globally in 2016 [2]. Low and middle-income countries (LMIC) accounted for 78% of all NCD deaths and 85% of premature adult NCD deaths worldwide [2]. The impact of NCDs on the global development agenda is well established, extending beyond Sustainable Development Goal (SDG) 3 on health and wellbeing to SDGs related to poverty, hunger, education, gender equality, and economic growth among others [3]. SDG target 3.4 specifies a one-third reduction in premature NCD mortality. Addressing health, lifestyle issues and social inequities that affect the emergence of NCD risk factors in individuals are essential in tackling NCDs, along with access to effective NCD treatments for those with an NCD [4–7].

The importance of access to safe, effective, quality and affordable medicines is reflected in SDG 3.8 and is underpinned by Universal Health Coverage (UHC) and financial risk protection for patients and their families. WHO’s Global Action Plan for the prevention and control of NCDs 2013–2020 (GAP) [1] includes a specific target on access to medicines, of ‘80% availability of the affordable basic technologies and essential medicines, including generics, required to treat major NCDs in both public and private facilities.’ The Independent High-Level Commission on NCDs has recommended that ‘governments should ensure that the national UHC benefit package includes NCD and mental health services … . as well as access to essential medicines and technologies.’ [8]

## Growing demands, unmet and unrecognized needs, limited financial resources: the perfect storm

The NCD burden is growing due to the increase in the common risk factors in parallel to an ageing population [1,9]. It is not just the increase of one NCD that poses a problem, but also the increase in multi-morbidity [10]. These epidemiological changes are occurring in health systems that are under-funded and not adapted to manage chronic diseases as well as provide access to affordable medicines.

A population-based study in Nicaragua estimated there should be 186,708 people with Type 2 diabetes. However, health system registries identified around 38,000 patients [11]. The diabetes population managed by the health system incurred costs representing 5% of the total budget of the Ministry of Health; managing all people with diabetes would require five times the resources. Similarly, in Mozambique, an estimated 33.1% of adults (5 million people) were hypertensive, with 14.8% (700,000 people) aware of having hypertension, 51.9% of those aware receiving treatment (350,000 people) and 39.9% of those treated being under control (150,000 people) [12]. These data highlight a variety of failures in the delivery of care for hypertension, but also the health system challenges should all these individuals be diagnosed, managed properly and medicines need to be addressed.

In the absence of UHC, patients and their families can face large out-of-pocket (OOP) costs for health care. High OOPs are often related to medicines [10,13]. In 2014, OOP expenditures in Nigeria were estimated at 71.1% of total health expenditure, compared to Venezuela 60%, Cameroon 66.3%, India 62.4%, the UK 9.7% and France 6.3% [14]. Total pharmaceutical spending in the outpatient sector as a percentage of total health expenditure ranges from 6.8% in Denmark to 29.2% in Hungary [15] with an OECD average of 16%. In the WHO Western Pacific Region, this ranged from 9.7% in New Zealand to 44% in Cambodia [16], underscoring the higher relative expenditure on medicines in LMICs [17].

## Priority setting and access to medicines for UHC

Budget constraints exist in all settings meaning that choices need to be made, thus priority-setting is inevitable and requires evidence-based, transparent processes based on national values and priorities, reflecting the concerns of the public and community at large [18]. This is particularly true for high-priced cancer medicines [19]. The essence of UHC is that out-of-pocket payments are not so high as to deter people from using services and causing financial hardship [20]. In parallel, the services need to be present to deliver the services people need.

In 2005, World Health Assembly resolution 58.33 committed governments to develop their health financing systems so that all people have access to services without suffering financial hardship paying for them [21]. However, progress has been impeded by lack of resources, over-reliance on formal and informal payments at the time of care and inefficient and inequitable use of resources [21]. Wirtz et al. [22] found that US$13 to US$25 per capita is needed to pay for a basic package of 201 essential medicines, but that current government expenditure on medicines in most LMICs is lower than this.

Both the range of medicines and extent of coverage/reimbursement of medicines under UHC are important. Health insurance systems in high-income countries (HIC) typically have a comprehensive package of medicines reimbursed. However, there is substantial variability. In the Asia-Pacific region, the number of products included in national EML, procurement and reimbursement lists ranges widely [16]. For example, in looking at products included on the reimbursement list, in the Republic of Korea 17,700 products are included, compared to 1,695 in Malaysia, 190 in Mongolia, more than 4,500 in New Zealand and 676 in the Philippines [16]. The outpatient health insurance medicines list in Kyrgyzstan is limited to 58 INNs, with patients still required to pay 50% of the medicines costs [23]. In Ukraine, the ‘Affordable Medicines’ program, implemented in April 2017 introduced reimbursement of some outpatient medicines (23 INNs) for patients diagnosed with either CVD, Type 2 diabetes, or asthma [24]. In Ghana, only some medicines for hypertension, diabetes, and cancers are included on the National Health Insurance Scheme [25]. Kyrgyzstan and Republic of Moldova have included a variety of NCD medicines in their package; however, availability of these was often poor [13].

In Europe, there is a complex mix of policies addressing medicine price regulation, generic substitution policies, positive medicines lists, differential reimbursement rates, patient financial ‘safety nets’, and varying protections for vulnerable groups [26]. Equally diverse are the payment methods for medicines in the public sector in Asian-Pacific countries, with Australia, New Zealand applying patient co-payments, full reimbursement for category A drugs in China with 70%-80% reimbursement for category B drugs, nominal user charges with exemptions for the poor in Cambodia, and capitation payments that include medicines in Indonesia [16].

China and Thailand offer examples on how LMICs can develop prioritization of medicines to include in their benefit packages. In China, the first step was the definition of the essential health package on the basis of cost, effectiveness, fairness, and affordability. Subsequently, financial resources were allocated to health and redevelopment of the social care model to ensure equity and effectiveness in providing financial protection were also part of this process [27]. Thailand uses health technology assessments to support decisions for including new medicines into the benefit package [28].

Quality issues for a variety of medicines are known: e.g. antimalarials and anti-infectives [29–32]; however, few studies look at this issue for NCD medicines. A study in Rwanda on anti-hypertensive medicines found that 20% of test formulations were of substandard quality at the time of purchase [33], and 70% were found to be of sub-standard quality after 6 months of testing in tropical climate conditions. A study in Africa of CVD medicines found a significant amount of poor-quality medicines in different contexts [34]. Besides the need to ensure quality medicines enter the health system, there also needs to be proper distribution, storage, prescription, and use in order to avoid wasting resources.

## The need for measurement and accountability

Standard methods, such as the WHO/Health Action International methodology, WHO Service Availability and Readiness Assessments surveys and studies such as Prospective Urban Rural Epidemiology [35] measure availability of medicines. Methods for assessing the affordability of medicines have included assessment of catastrophic expenditure (if it exceeds 5% of daily income), household impoverishment (if the residual income after purchasing medicines was less than US$1.25 or US$2 per day), and estimates based on the salary of the lowest paid government worker (LPGW), that proposes that a month’s treatment should not cost more than 1 day of wages [36,37]. More recently, WHO has proposed a measure where ‘a medicine is affordable when no extra daily wages (EDW) are needed for the lowest paid unskilled government sector worker (LPGW wage) to purchase a monthly dose treatment of this medicine after fulfilling basic needs represented by the national poverty line (NPL)’ [38].

These different study approaches have shown variation in estimates [39] as well as providing a snapshot versus ongoing systematic assessment of availability over time using monitoring and evaluation systems. Monitoring and evaluation of the implementation of UHC as well as measuring access to medicines (including availability and affordability) are key components of sustainable systems [22,40,41] as is the need for a system of accountability [42]. This would require political support, national capacity strengthening, use innovations for better data collection and analysis and advocacy.

## Discussion

Once diagnosed with an NCD the individual faces many hurdles in accessing treatment. In LMICs this is due to various access issues, health system capacity and where NCD care is delivered. HICs also face this challenge, again related to adequate use of resources, e.g. specialist care versus primary care and cost-effective medicines in a context of increasing NCD, multi-morbid and complex patients.

Although the settings are different the approaches needed to ensure access to medicines in the context of UHC are similar in both LMICs and HICs. In both a comprehensive approach is needed in strengthening health systems and the full medicine supply chain. In LMICs it could be argued that the challenges are more in terms of allocation of resources for NCD medicines and procurement and supply systems. To date, many of the initiatives aiming to improve access to NCD medicines in LMICs have been driven by the pharmaceutical industry [43]. While these programs can facilitate access, they do not replace the need for long-term commitments by governments to comprehensive system changes that will underpin sustainable and affordable access to NCD medicines. As many LMICs ‘graduate’ from being reliant on donor assistance there is the need to strengthen a variety of elements of their health system from procurement systems to healthcare delivery in a context of increasing NCD burden, UHC and less external financial resources [44]. In addition, a focus on four main NCDs (CVD, diabetes, CRD, and cancer) by the global health community may detract prioritization for access to medicines for other NCDs.

Whilst availability is not a problem in HICs issues around prescribing, dispensing, use, health budgets and in some cases incomplete coverage and high prices are challenges for affordability [45]. For example, despite new medicines and increasing costs for diabetes management, clinical outcomes have not followed suit [45]. The drivers of the challenge in LMICs is that of increasing numbers in settings where there are weak health systems, whereas in HICs it is the increasing cost of medicines in a context where health systems are not adapted to managing the chronic nature of NCDs in parallel to a lack of focus on risk factors and their determinants.

The availability and affordability of medicines for NCDs remain key concerns, but are amenable to action. An overall policy and governance framework for medicines is needed that includes addressing regulatory issues to ensure the quality of medicines in circulation, along with greater use and acceptance of generic medicines to support access to affordable medicines for patients and to help contain medicines costs for health-care systems [41]. Priority NCD medicines should be included in benefits packages, supported by price regulation and efficient procurement and distribution of these medicines [41] in conjunction with ongoing monitoring systems. Data generated should be actively used both globally and nationally to inform and monitor progress as well as for accountability purposes. Money and medicines alone are not sufficient as meeting the demands of NCDs requires a coordinated and comprehensive health system response tackling complex issues of financing, the health workforce, information, medicines policies, and change management strategies to upscale cost-effective interventions for both prevention and treatment of NCDs [7].

## Paper context

Universal Health Coverage is indispensable to reach Sustainable Development Goal 3, and access to essential medicines is a critical component. Increasing prevalence of noncommunicable diseases, along with under-diagnosis and under-treatment, in parallel to increasing costs of noncommunicable disease medicines pose a threat to Universal Health Coverage in all settings. In this paper these challenges are described and proposals to develop appropriate responses to guarantee equitable access presented.
